# COVID-19 Pneumonia: The Great Ultrasonography Mimicker

**DOI:** 10.3389/fmed.2021.709402

**Published:** 2021-08-25

**Authors:** Donato Lacedonia, Carla Maria Irene Quarato, Antonio Mirijello, Guglielmo M. Trovato, Anna Del Colle, Gaetano Rea, Giulia Scioscia, Maria Pia Foschino Barbaro, Marco Sperandeo

**Affiliations:** ^1^COVID-19 Center, Department of Medical and Surgical Sciences, Institute of Respiratory Diseases, Policlinico Universitario “Riuniti” di Foggia, University of Foggia, Foggia, Italy; ^2^COVID-19 Unit, Department of Internal Medicine, Istituto di Ricovero e Cura a Carattere Scientifico (IRCCS) Fondazione Casa Sollievo della Sofferenza, San Giovanni Rotondo, Italy; ^3^The European Medical Association, Brussels, Belgium; ^4^Department of Radiology, Azienda Ospedaliera dei Colli-Cotugno and Monaldi Hospital, Napoli, Italy; ^5^Unit of Interventional and Diagnostic Ultrasound, Department of Internal Medicine, Istituto di Ricovero e Cura a Carattere Scientifico (IRCCS) Fondazione Casa Sollievo della Sofferenza, San Giovanni Rotondo, Italy; ^6^Professor of Diagnostic and Interventional Lung Ultrasonography at the Bachelor in Medicine and Surgery and the Postgraduate School of Respiratory Disease, University of Foggia, Foggia, Italy

**Keywords:** lung ultrasound, chest computed tomography, COVID-19 pneumonia, COVID-19 pneumonia mimickers, specificity

## Abstract

The pandemic spread of the new severe acute respiratory syndrome coronavirus 2 has raised the necessity to identify an appropriate imaging method for early diagnosis of coronavirus disease 2019 (COVID-19). Chest computed tomography (CT) has been regarded as the mainstay of imaging evaluation for pulmonary involvement in the early phase of the pandemic. However, due to the poor specificity of the radiological pattern and the disruption of radiology centers' functionality linked to an excessive demand for exams, the American College of Radiology has advised against CT use for screening purposes. Lung ultrasound (LUS) is a point-of-care imaging tool that is quickly available and easy to disinfect. These advantages have determined a “pandemic” increase of its use for early detection of COVID-19 pneumonia in emergency departments. However, LUS findings in COVID-19 patients are even less specific than those detectable on CT scans. The scope of this perspective article is to discuss the great number of diseases and pathologic conditions that may mimic COVID-19 pneumonia on LUS examination.

## Introduction

The pandemic spread of the new severe acute respiratory syndrome coronavirus 2 (SARS-CoV-2) has solicited the necessity to identify a diagnostic method to early detect coronavirus disease 2019 (COVID-19). The reference standard test for diagnosing SARS-CoV-2 infection is real-time reverse-transcription-polymerase (RT-PCR) on nasopharyngeal swabs. The discriminating parameter for which a patient with suspected COVID-19 is hospitalized or sent home for follow-up is the presence of an even subclinical respiratory failure that is linked to the possibility of pulmonary involvement. Functional data can be easily obtained with the measurement of peripheral saturation (SpO_2_), the execution of a blood gas analysis, or with a 6-min walking test although the anatomical-morphological data derive from chest imaging.

A standard chest X-ray (CXR) is characterized by low sensitivity in identifying the earliest pulmonary changes in COVID-19 (1, 2). On the contrary, a chest computed tomography (CT) scan is more accurate in the study of initial ground glass opacities (GGOs) (2). For this reason, chest CT has been used in the early period of strong emergency in China (especially in the Hubei area) as the imaging of choice for the assessment of patients with SARS-CoV-2 infection. However, COVID-19 pneumonia exhibits varied and non-specific high-resolution CT (HRCT) features, mimicking other lung infections (e.g., influenza A; other coronavirus, including severe acute respiratory syndrome coronavirus and Middle East respiratory syndrome coronavirus; cytomegalovirus and atypical germs, including chlamydia and mycoplasma) or closely resembling imaging findings of several other non-infectious pathologies [e.g., pulmonary edema, pulmonary hemorrhage and infarction, pulmonary proteinosis, organizing pneumonia (OP), sarcoidosis, interstitial lung diseases (ILDs), and neoplasms] (3). Furthermore, during the 1st days of infection a negative CT cannot exclude the possibility of COVID-19 (4). Therefore, also to avoid the disruption of radiology centers' functionality due to an excessive demand for exams, the American College of Radiology stated that CT in COVID-19 was not to be used for screening purposes, suggesting its employment only in hospitalized patients, symptomatic or with specific clinical indications (5). In other words, chest CT has an incremental diagnostic value only when it can have an effective impact on the management and the underlying therapy of COVID-19 patients (6).

In a clinical setting, such as that of COVID-19 pneumonia, bedside lung ultrasound (LUS) has an undeniable number of advantages, such as fast availability, ease of disinfection, and the possibility to perform the examination without moving patient, thus reducing the risk of spreading the virus while respecting the appropriate protective measures. Therefore, numerous authors suggest the use of LUS as a first approach for diagnosis of COVID-19 pneumonia in the emergency department (ED) (7–9). On the other hand, however, LUS disadvantages include the impossibility of exploring the whole lung parenchyma and a low specificity. A recent Cochrane's meta-analysis calculated a specificity of only 45% for LUS (10).

The scope of this perspective article is to discuss the great number of diseases and pathologic conditions that may exhibit ultrasound findings similar to those found in COVID-19 patients.

### COVID-19 Pneumonia Findings on Chest Imaging

COVID-19 presentation on chest imaging changes according to the stage of the disease (Table 1).

**Table 1:** Comparison between findings of different stages of COVID-19 pneumonia on Chest X-ray, CT scan, and LUS.

**Phases**	**Chest CT**	**Chest X-ray**	**Lung ultrasound**
Early	May be negative or with focal GGOs	Usually negative	Focal B-lines
Mild	Peripheral confluent and bilateral GGOs	May be negative or with few reticular opacities	Focal B-lines with thickened pleural line
Moderate	“Crazy paving” pattern	Reticular opacities mixed with prevalent hazy lung opacities	Confluent B-lines with thickened pleural line
Severe	Lung consolidations	Diffuse coalescent lung opacities and/or consolidative pattern	Lung consolidations (parenchymal hepatization)

Although chest CT may be normal in the first 5 days of infection (4), during the early phase of the disease, there is a predominance of focal areas of GGOs with peripheral distribution in one or both lungs. Mild pneumonia is still characterized by a predominance of GGOs. However, there may be a higher number of such opacities and bilateral involvement. In moderate disease, there may also be a superimposing inter- and intralobular septal thickening, resulting in the so-called “crazy paving” pattern (11). Finally, patients with severe pneumonia usually show peripheral consolidations in a bilateral distribution (12).

CXR is certainly less sensitive than a CT scan in revealing the typical GGOs of early and mild disease. When detectable, early alterations on CXR are predominantly reticular, and hazy lung opacities are predominantly described in an intermediate phase of the disease. In the late phase, lung opacities may evolve into a diffuse, coalescent, or consolidative pattern. Lesions are generally bilateral and tend to have a lower lung distribution, mirroring what is noticeable on chest CT. Severe lung disease usually involves the majority of the pulmonary parenchyma (13).

Regarding LUS examination, the first pulmonary manifestation consists in the presence of ≥3 focal vertical artifacts (B-lines). In mild pneumonia, the finding of ≥3 focal B-lines is associated with a thickened and irregular pleural line. Subsequently, in moderate pneumonia, B-lines tend to become confluent and are associated to a thickened and more irregular pleural line. In severe pneumonia, lung lesions can evolve in areas of consolidation, especially in a gravitational position (7–9). These ultrasound findings are regarded as suggestive of a progressive loss of aeration as the severity of the disease increases (9, 14).

### Lung Ultrasound Technique: Artifacts and Pitfalls

As the normal aired lung reflects almost all of the ultrasound beam (more than 95%), LUS imaging is mainly based on the evaluation of the so-called hyperechoic “pleural line” and other reverberation artifacts, classifiable as horizontal A-lines or vertical B-lines. Although the presence or not of these artifacts can be suggestive of a disease condition, they are essentially “imaging errors” due to the great difference in acoustic impedance between soft tissues of the chest wall and the pulmonary air content (15, 16).

**Figure 1:**
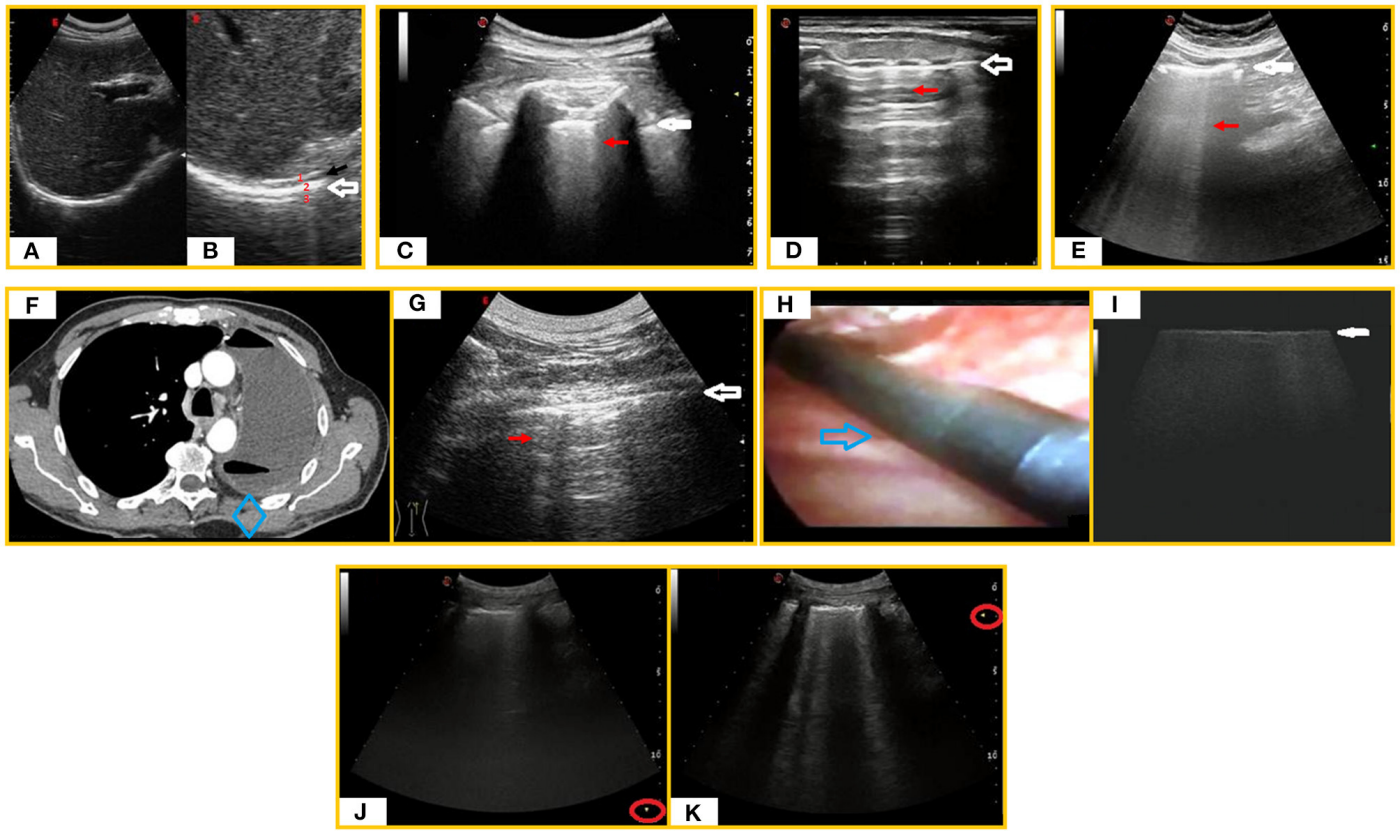
LUS artifacts. **(A)** Subcostal right ultrasound (US) scan with a convex probe (6 MHz). **(B)** Magnification on the “diaphragmatic hyperechoic line” (white arrow) showing that it is actually composed of three hyperechoic lines (1. hepatic capsule/diaphragmatic muscle interface line, 2. diaphragmatic muscle/pulmonary base interface line, 3. mirror reflection artifact) within which the real diaphragmatic muscle appears as a thin hypoechoic line (black arrow). **(C)** LUS scan with a convex probe (6 MHz) in a healthy individual showing the “hyperechoic pleural line” (white arrow) followed by a sporadic B-line (red arrow). **(D)** Longitudinal tracheal US scan with a linear probe (8 MHz) showing a “tracheal hyperechoic line” at the soft tissue/tracheal air interface (white arrow) followed by reverberation artifacts (red arrow). **(E)** Abdominal US scan with a convex probe (6 MHz) showing a “hyperechoic peritoneal line” (white arrow) followed by laser-like vertical and confluent ring down artifacts (red arrow). **(F)** Axial chest CT scan in a post-pneumonectomy patient revealing air and fluid collection in the residual space with mediastinal sliding. The intact lung is shifted toward the residual cavity. **(G)** US scan of the post-pneumonectomy space [corresponding to the blue box in the **(F)** CT scan] revealing a hyperechoic interface line (white arrow) followed by reverberation artifacts (red arrow). **(H)** Image of the pulmonary parenchyma of a patient diagnosed with usual interstitial pneumonia UIP during VATS. A sterile intracavitary laparoscope probe (12 MHz) with 10-mm diameter and 38-cm length (blue arrow) was introduced through one of the VATS ports in the thoracic cavity. **(I)** Intraoperatory LUS scan (linear probe, 12 MHz) showing an irregular increased thickness of the pleura line (white arrow) with no artifact below it. **(J,K)** LUS scans showing how modification of focus position (red round) can modify the number of vertical artifacts.

The hyperechoic pleural line itself is a virtual image that has no real anatomical correlation. Indeed, the actual thickness of the whole pleural membrane, including its visceral and parietal sheets and the virtual space between them, measures ~120–140 μm under the microscope (17). Similarly, the diaphragm appears as a hyperechoic line above the ipsilateral lung base. However, this artifact is actually due to a physical interaction of ultrasound resulting in the production of three hyperechoic lines, within which the real hypoechoic diaphragmatic muscle corresponds to a thin hypoechoic line (Figures 1A,B).

An increase in B-lines may also be found in healthy individuals, generally at the bases, where the hydrostatic pressure gives a more fluid-rich interstitium (18) (Figure 1C). In case of tracheitis, due to the alteration in the proportion between the tracheal air and the interstitial fluid content, an ultrasound longitudinal tracheal scan may show a hyperechoic line at the soft tissue/tracheal air interface with underlying reverberation artifacts (Figure 1D). On abdominal ultrasound, it is also possible to identify laser-like vertical, focal, or confluent hyperechoic artifacts, arising from a hyperechoic peritoneal surface and reaching the border of the scan field, mimicking lung B-lines (19, 20). This ultrasound pattern may be simply indicative of a normal amount semifluid fecal material within air-filled bowel loops (Figure 1E). Also in post-pneumonectomy patients, LUS shows a fixed hyperechoic line at the chest wall–residual cavity interface; this ultrasound finding can even be accompanied by ring-down artifacts when the ultrasound beam passes through a mixture of liquid and air, such as that sometimes forming in the residual post-pneumonectomy cavity (21) (Figures 1F,G). On the contrary, generation of B-line artifacts does not occur in the intra-operatory ultrasound examination of fibrotic lung during video-assisted thoracoscopic surgery (VATS-US) despite the presence of B-lines in transthoracic LUS. Indeed, the VATS-US approach is not limited by differences in acoustic impedance because the ultrasound probe is directly in contact with the lung (22) (Figures 1H,I).

The appearance and number of ultrasound artifacts is strongly influenced by the type and frequency of probe used, the time gain compensation (TGC), the lack of tissue harmonics, and the focus of the ultrasound beam. Therefore, one of the main hindrances to the reproducibility of LUS examination is the machine setting. High-frequency linear probes (8–12.5 MHz) improve spatial resolution and reduce the number of ultrasound artifacts but allow a shallower depth of penetration. Low-frequency convex probes (3–8 MHz) have greater depth of penetration but generate lower spatial resolution, increasing the number of artifacts. For instance, the thickness of the pleural line of the same healthy subject can measure 0.7–1.8 mm using a high-frequency probe (8–12.5 MHz) and 1.4–2.8 mm with a middle- to low-frequency probe (3.5–5 MHz). Sectorial cardiac probes (2–3.5 MHz) create a folding in the ultrasound beam that generates a higher number of B-lines. Similarly, excessive TGC and the lack of tissue harmonic imaging are generally associated with the detection of a higher number of vertical artifacts. TGC should not exceed 50%, and tissue harmonics are preferable. It is also important to position the focus at the level of the pleural line to prevent misinterpretations of the vertical artifacts (23) (Figures 1J,K).

To properly explore the 70% of the pleural surface that is not hidden by the bony structures of the rib cage, LUS should be performed with the patient in a sitting or semi-sitting position. In emergency settings and in cases of critical patients, the examination can be performed in supine and lateral positions. The whole lung fields should be examined from the bases up to the ipsilateral apexes with longitudinal and transversal intercostal scans along the anatomical lines of the thorax. Posteriorly, we can opt for longitudinal and transversal intercostal scans along the para-vertebral and mid-scapular lines. Laterally, we can use the posterior- and mid-axillary lines. Anteriorly, chest scans can be performed along the anterior-axillary, mid-clavicular, and parasternal lines (15, 16).

Finally, it should be underlined that the simple change of positioning of the probe with respect to the curvature of the patient's chest and the patient's respiratory rate may increase the perceived occurrence of B-lines (15, 23, 24). This increases the risk of inter- and intraoperative variability and bias in the case of highly dispnoic patients.

## Sonographic Covid-19 Pneumonia Mimickers

### Cardiogenic Pulmonary Edema (CPE)

**Figure 2:**
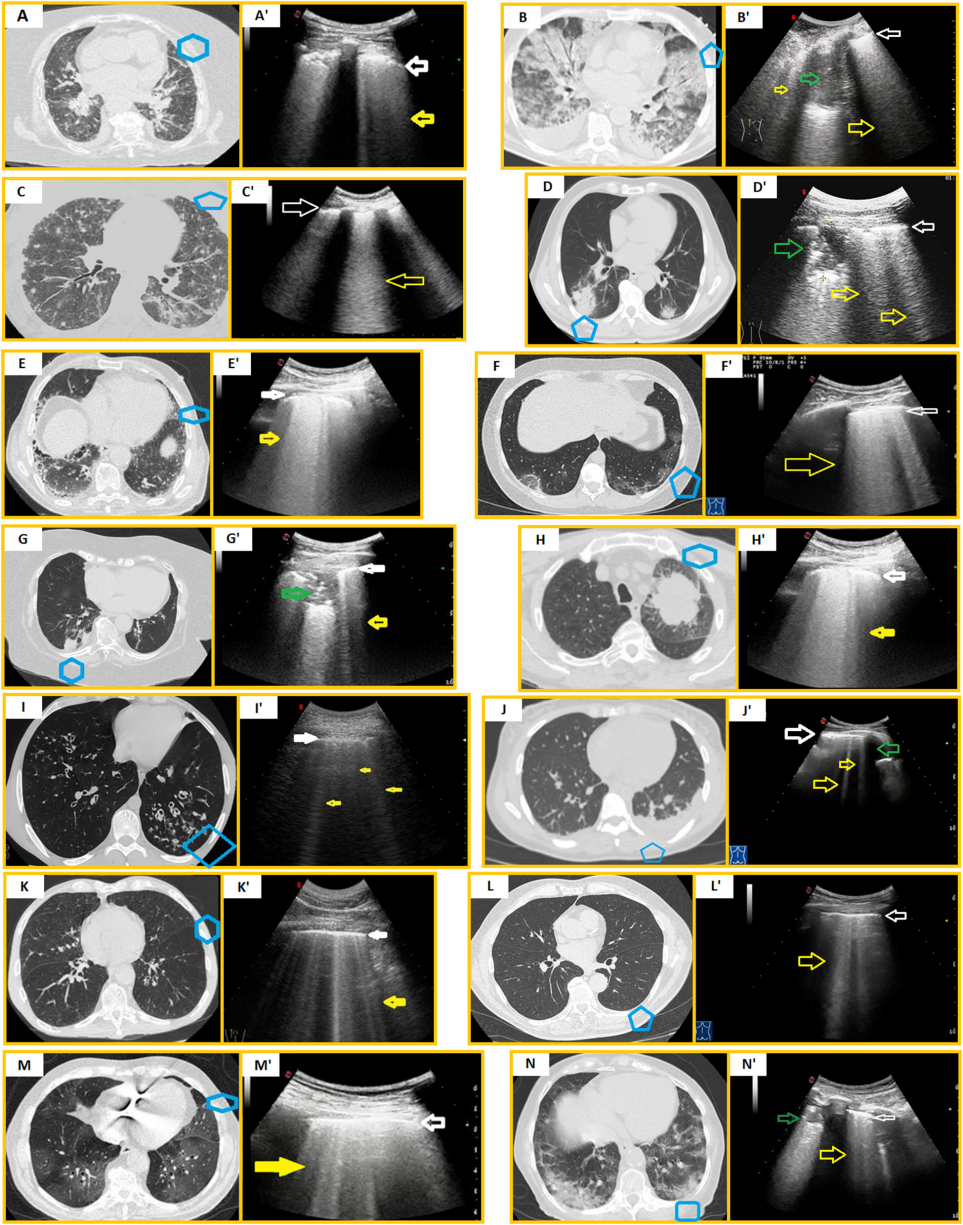
Mimickers of COVID-19 pneumonia vs. COVID-19 pneumonia. Blue boxes in CT scans indicate the corresponding US scans. **(A)** Axial chest HRCT of a CPE. **(A')** US scan with a convex probe (6 MHz) shows a blurred and thickened hyperechoic pleural line (white arrow) followed by focal and coalescent B-lines (yellow arrow). **(B)** Axial chest HRCT scan of ARDS. **(B')** US scan with a convex probe (6 MHz) shows a thickened hyperechoic pleural line (white arrow) followed by coalescent B-lines (yellow arrows). A hypoechoic consolidation (green arrow) is also viewable. **(C)** Axial chest HRCT scan of CMV pneumonia. **(C')** US scan with a convex probe (6 MHz) shows a blurred and thickened hyperechoic pleural line (white arrow) followed by coalescent B-lines (yellow arrow). **(D)** Axial chest HRCT scan of bacterial pneumonia. **(D')** US scan with a convex probe (6 MHz) shows a mixed hypo-hyperechoic lesion (green arrow) and a thickened hyperechoic pleural line (white arrow) followed by coalescent B-lines (yellow arrows). **(E)** Axial chest HRCT scan of an idiopathic pulmonary fibrosis. **(E')** US scan with a convex probe (6 MHz) shows a thickened hyperechoic pleural line (white arrow) followed by coalescent B-lines (yellow arrow). **(F)** Axial chest HRCT scan of OP (areas of ground glass, mild reticular pattern, and “reversed halo sign”). **(F')** US scan with a convex probe (6 MHz) shows a thickened hyperechoic pleural line (white arrow) followed by coalescent B-lines (yellow arrow). **(G)** Axial chest CT scan of a poorly differentiated lung carcinoma (histological diagnosis). **(G')** US scan with a convex probe (6 MHz) shows a mixed hypo-hyperechoic lesion (green arrow) interrupting the pleural line's continuity (white arrow) with an adjacent B-line (yellow arrow). **(H)** Axial chest HRCT scan of a lung adenocarcinoma (histological diagnosis). The neoplastic mass does not adhere to the pleural surface. **(H')** US scan with a convex probe (6 MHz) shows a thickened pleural line (white arrow) with coalescent B-lines below it (yellow arrow). **(I)** Axial chest HRCT scan showing bronchiectasis **(I')** US scan with a convex probe (6 MHz) shows a thickened pleural line (white arrow) followed by numerous B-lines (yellow arrows). **(J)** Axial chest HRCT showing pleural effusion. **(J')** US scan with a convex probe (6 MHz) shows a thickened pleural line (white arrow) followed by an anechoic pleural effusion (green arrow) and focal discrete B-lines (yellow arrows). **(K)** Axial chest HRCT showing COPD exacerbation. **(K')** US scan with a convex probe (6 MHz) shows a thickened pleural line (white arrow) followed by numerous B-lines (yellow arrow). **(L)** Axial chest HRCT showing signs of aging lung (increased broncho-arterial ratio, age-related alveolar hyperinflation, and a thoracic spine osteophyte). **(L')** US scan with a convex probe (6 MHz) shows a thickened pleural line (white arrow) followed by several B-lines (yellow arrow). **(M)** Axial chest HRCT scan of a moderate COVID-19 pneumonia. **(M')** US scan with a convex probe (6 MHz) shows a thickened pleural line (white arrow) followed by numerous and also coalescent B-lines (yellow arrow). **(N)** Axial chest HRCT scan of a severe COVID-19 pneumonia. **(N')** US scan with a convex probe (6 MHz) shows an unspecific mixed hypo-hyperechoic consolidation (green arrow) and a thickened hyperechoic pleural line (white arrow) followed by numerous B-lines (yellow arrow).

The initial event in CPE is left ventricular dysfunction. The reduced cardiac output, in conjunction with excessive end-diastolic filling pressure, lead to raised pressures in the left atrial, pulmonary veins, and capillary, allowing protein-poor fluid to traverse the capillary membranes into the pulmonary interstitium and alveolar air spaces. Distinguishing between COVID-19 pneumonia and CPE may be challenging. On LUS, fluid-filled secondary interlobular septa act as acoustic traps in which incident ultrasound waves reverberate, giving rise to an unspecific pattern of discrete and uniform vertical B-lines, which is observable also in early COVID-19 pneumonia. As the secondary interlobular septa are more distended by the transudative fluid, discrete B-lines tend to assume a confluent appearance, resembling moderate and severe COVID-19 disease. Despite in CPE the hyperechoic pleural line maybe appearing as blurred and thickened, it is generally more regular in shape compared with COVID-19 pneumonia (25). However, these subtle differences cannot be easily recognized by an inexperienced eye. Bilateral and symmetrical GGOs in the dependent regions of the lower lobes and interlobular septal thickening represent overlapping features on chest CT although a peri-hilar distribution of GGOs is more typical for CPE (26, 27). The presence of bilateral pleural effusion and cardiomegaly may help orienting toward a diagnosis of CPE (28). Anyhow, it should be pointed out that COVID-19 pneumonia and CPE may also coexist (29) (Figures 2A,A').

### Acute Respiratory Distress Syndrome (ARDS)

In ARDS, the damage of the alveolar-capillary membrane causes a heterogeneous exudative edema. Part of the lung volume, which was originally occupied by air, may be replaced with fluid, connective tissue, cells, and/or hyaline membrane, creating acoustic traps for the ultrasound beam and, consequently, an ultrasound pattern of focal or coalescent B-lines. The pleural line appears frequently irregular, thickened, and coarse because of the presence of multiple small subpleural consolidations (30) (Figures 2B,B'). CT features are confluent GGOs and air–space consolidations representing edema and protein within the interstitial and alveolar spaces (31). Because COVID-19 pneumonia itself represents a cause of ARDS in the most severe stages (32), the differential diagnosis from the other causes of alveolar damage is practically impossible on the basis of chest imaging and in the absence of viral testing.

### Viral Pneumonia

Viral pneumonia, such as influenza A virus (H7N9, H1N1), respiratory syncytial virus, parainfuenza virus, adenovirus, cytomegalovirus (CMV), and human metapneumovirus (33–36), commonly manifests as a sonographic “alveolar-interstitial syndrome” with unspecific ultrasound findings, including ≥3 focal or confluent B-lines, a thickened and irregular hyperechoic pleural line, and subpleural consolidations (Figures 2C,C'). Therefore, the ultrasound pattern of other viral pneumonia may practically mimic that of all stages of COVID-19. Specific diagnosis requires viral tests.

### Bacterial Pneumonia

Bacterial pneumonia is detectable on LUS examination only when it involves the peripheral parenchyma facing to the superficial pleura. Community-acquired pneumonia is usually accessible to LUS due to the frequent involvement of the pleuropulmonary surface (in about 70–80% of cases) (37). LUS appearance of pneumonia is a hypo-anechoic area with poorly defined margins, especially in depth (37, 38). Within the lesion, in a percentage of about 50%, hyperechoic or hypo-anechoic spots and/or striae may be detected (37). Pleural effusion is frequently associated (37, 38) (Figures 2D,D'). Although pleural effusion is an atypical finding in COVID-19 pneumonia, the ultrasound pattern of subpleural consolidations is highly unspecific and does not allow a certain differential diagnosis between COVID-19 and bacterial pneumonia. Furthermore, bacterial overlaps are not uncommon in patients with severe COVID-19 pneumonia or prolonged hospitalization for COVID-19 (39).

### ILDs and OP

LUS signs in ILDs are (1) a thickened hyperechoic pleural line (>3.0 mm with convex probe), (2) an irregular and/or fragmented and/or blurred aspect of the hyperechoic pleural line, (3) an increase in the number (≥3) of B-lines between two ribs in a single scan, (4) evidence of subpleural nodulations (40, 41). The measurement in millimeters of the hyperechoic pleural line shows a direct correlation with increasing degree of lung involvement on HRCT scans (40, 42) (Figures 2E,E'). Furthermore, pleural line irregularities appear more pronounced in the areas of greater fibrotic alteration (40). B-lines in ILDs are generated by an increased density of the lung due to a decrease in the amount of air and/or an increase in interstitial tissue. These phenomena generate channels of acoustic permeability for the ultrasound beam as a consequence of the reduction in the difference of acoustic impedance between chest wall tissues and aerated lung. As a further confirmation, on intra-operatory VATS-US examination of fibrotic lung (when the probe is directly in contact with the pulmonary parenchyma), we can assess only an increased thickness of the pleura line without vertical artifacts below it (22).

The ultrasound appearance of ILDs is practically superimposable to that of COVID-19 pneumonia. Moreover, distinguishing between pulmonary abnormalities caused by COVID-19 and some types of interstitial pneumopathy may be very challenging even on CT scan. The differential diagnosis appears particularly difficult for non-infectious OP (43) (Figures 2F,F').

### Neoplasms, Central Atelectasis, and Pulmonary Lymphangitic Carcinomatosis (PLC)

The ultrasound appearance of peripheral lung neoplasms is that of solid lesions of variable nodular or oval shape with regular or irregular margins. Their echo pattern may be hypoechoic or mixed hypo-hyperechoic due to the presence of hyperechoic striae or spots inside (Figures 2G,G'). Sometimes neoplasms can also show an anechoic pattern, mimicking the appearance of fluid collections (e.g., lung abscesses). To date, there is no ultrasound pattern characterizing subpleural lung masses (15, 16). As a result, LUS does not discriminate between subpleural lung cancer and pneumonia, including COVID-19. Sometimes, airway obstruction due to a primary intraluminal neoplasm, airway metastasis invasion, or extrinsic compression may occur, giving rise to a resorptive atelectasic area. Obstructive atelectasis has the same ultrasound appearance of other lung consolidations (i.e., hypoechoic or mixed hypo-hyperechoic), including COVID-19 pneumonia. However, atelectasis is detected only if it extends to the pleura. Conversely, an area of peripheral atelectasis or any consolidation that does not reach the pleural surface may cause a non-specific increase in B-lines as a result of the variation of the normal proportional content between air, lung tissue, and fluid (Figures 2H,H'). This gives rise to another possible false positive sonographic pattern of early COVID-19 pneumonia.

PLC consists of an inflammation of the lymphatic vessels (lymphangitis) associated with a malignancy. It may be caused by the dissemination of tumoral cells along the lymphatics or by the block of lymphatic drainage from a centrally located mass. Eighty percent of PLC are from adenocarcinomas (44). LUS examination may reveal a thickened and fragmented hyperechoic pleural line with an increased number of B-lines (≥3) and subpleural nodulations (45). As a result, PLC represents another mimicker of COVID-19 pneumonia at LUS.

### Bronchiectasis

Bronchiectasis consists of an irreversible structural change of lung airways, including bronchial dilatation, bronchial wall thickening, and formation of mucus plugs due to impaired drainage of bronchial secretions. A chronic infection of accumulated viscous secretions may occur, stimulating and sustaining lung inflammation with an increased frequency of exacerbations, finally resulting in rapid lung function decline (46). LUS findings may include a normal aired lung condition, an increased number of B-lines, or a consolidative pattern, depending on the type (i.e., cylindrical, varicose, and cystic) and extent of bronchiectasis valuable on the HRCT scan (47) (Figures 2I,I'). Therefore, bronchiectasis may simulate all the degrees of COVID-19 pneumonia severity on LUS examination.

### Pleural Effusion

An important role of ultrasound in the lung is the possibility to discover pleural effusion, which sometimes is not visible on standard chest radiographs, especially if minimal (<10 ml) and associated with basal consolidations (e.g., pneumonia or neoplasms). Pleural effusion is generally associated with multiple vertical, discrete, or confluent B-lines, arising from the deeper side of the fluid collection (Figures 2J,J'). The finding of atelectasic areas resulting from the compressive effect on the lung parenchyma by pleural effusion is also quite frequent. In these cases, the atelectatic lung is visualized below the effusion. In the presence of these findings, the exclusion of COVID-19 pneumonia is not possible on the basis of the ultrasound patter alone.

### Emphysema and Acute Chronic Obstructive Pulmonary Disease (COPD) Exacerbations

In COPD patients have an irregular and thickened pleural line that may be related to smoking-induced remodeling of lung parenchyma and subpleural areas of emphysema. COPD exacerbations may be also associated with an increased number of B-lines (48). Given the presence of suggestive respiratory symptoms, it is difficult to rule out COVID-19 pneumonia *a priori* (Figures 2K,K').

### Lung Contusions

The sonographic pattern indicative of lung contusion includes an increase in B-lines artifacts associated or not to the presence of hypoechoic or mixed hypo-hyperechoic subpleural focal consolidations (49), configuring another possible mimicker of COVID-19 pneumonia.

### Aging Lung

Some authors have evidenced that a thickened pleural line and an increase in B-lines may be related to an aging lung (Figures 2L,L') (50). It is, therefore, virtually impossible to rule out COVID-19 pneumonia using LUS in the elderly.

## Conclusions

LUS findings suggestive of COVID-19 pneumonia are clearly non-specific, being detectable in a large number of other infectious and non-infectious diseases (Figures 2M,M', N,N'). Despite LUS positive predictive value maybe being high in the setting of the SARS-CoV2 pandemic, the risk of a misdiagnosis increases when the prevalence of COVID-19 decreases, the patient's RT-PCR result is negative, or there is an underlying disease. The awareness of the impossibility of discriminating between some preexisting or supervening pathological conditions based on LUS alone is essential to avoid the dissemination of erroneous and potentially dangerous information. Integration between a patient's clinical background, laboratory tests, and more accurate radiological assessments is needed for differential diagnosis.

## Data Availability Statement

The original contributions presented in the study are included in the article/supplementary material, further inquiries can be directed to the corresponding author/s.

## Ethics Statement

Due to the descriptive nature of this manuscript, a written informed consent to participate in a clinical study was not required. The explanatory pictures included in this manuscript are from examinations performed as part of our routine medical practice for which patients signed informed consent. Patients provided informed permission for the image publication. The images have been anonymized to protect patients' privacy.

## Author Contributions

MS, DL, and CQ designed and directed the work. All the authors contributed to writing the manuscript, revised it critically, and read and approved the submitted version.

## Conflict of Interest

The authors declare that the research was conducted in the absence of any commercial or financial relationships that could be construed as a potential conflict of interest.

## Publisher's Note

All claims expressed in this article are solely those of the authors and do not necessarily represent those of their affiliated organizations, or those of the publisher, the editors and the reviewers. Any product that may be evaluated in this article, or claim that may be made by its manufacturer, is not guaranteed or endorsed by the publisher.
